# 

**Published:** 1902-03

**Authors:** 


### THE BRISTOL

### Medico-Chirurgical Journal.

A JOURNAL OF THE MEDICAL SCIENCES FOR THE WEST OF ENGLAND AND SOUTH WALES

*PUBLISHED UNDER THE AUSPICES OF*

*THE BRISTOL MEDICO-CHIRURGICAL SOCIETY.*

EDITOR:
R. SHINGLETON SMITH, M.D., B.Sc.,

WITH WHOM ARE ASSOCIATED
J. MICHELL CLARKE, M.A., M.D., J. LACY FIRTH, M.S., M.D.,
AND JAMES SWAIN, M.S., M.D.

ASSISTANT-EDITOR:
P. WATSON WILLIAMS, M.D.

EDITORIAL SECRETARY:
JAMES TAYLOR.

"Scire est nescire, nisi id me

Scire alius sciret."

LIBRARY

SURGEON GENERAL'S OFFICE

JUL. 14. 1903

182304.

*VOL. XX.*

BRISTOL: J. W. ARROWSMITH.
LONDON: J. & A. CHURCHILL, 7 GREAT MARLBOROUGH STREET.
1902.

W1
BR323

J.W. Arrowsmith Publisher Bristol

33754

### CONTENTS OF VOLUME XX.

Page

The Reputation of the Hotwells (Bristol) as a Health-Resort. By L. M. Griffiths, M.R.C.S. ... ... 1, 142, 193

On some Urinary Infections, with Especial Reference to their Treatment by Urotropin. By J. Odery Symes, M.D., D.P.H. 27

A Retrospect of a Further Series of Intra-Abdominal Operations. By James Swain, M.S., M.D. Lond., F.R.C.S. ... ... ... 33

The Use of the X-Rays in the Diagnosis of Renal Calculi. By James Taylor, M.R.C.S. ... ... ... ... ... ... 44

Notes on a Case of Variola Nigra. (Malignant or Black Smallpox.) By D. S. Davies, M.D. ... ... ... ... ... ... 49

Notes on Ethyl Chloride as a General Anæsthetic. By Frank H. Rose, M.R.C.S., L.R.C.P. Lond. ... ... ... ... 53

A Case of Gastro-Jejunostomy for Cancer of the Pylorus. By J. Paul Bush, C.M.G., M.R.C.S. ... ... ... ... ... 57

Edward Long Fox, M.D. Oxon, F.R.C.P. ... ... ... ... 97

Bonville Bradley Fox, M.A., M.D. Oxon. ... ... ... ... 105

The Diagnosis of Oriental or Bubonic Plague. By E. Klein, M.D., F.R.S. ... ... ... ... ... ... ... ... 108

Two Cases of Localised Necrosis of the Lung, with Recovery. By J. Michell Clarke, M.A., M.D., F.R.C.P. ... ... ... 122

Some Cases of Ptosis. By W. M. Beaumont, M.R.C.S., L.S.A. ... 131

Diplococcal Bronchitis. By P. Watson Williams, M.D. Lond.... 135

A Case of Ascites due to Thrombosis of the Hepatic Veins, By Theodore Fisher, M.D., M.R.C.P. ... ... ... ... ... 209

Paralysis of the Accommodation—An *A Posteriori* View of Diphtheria. By W. M. Beaumont, Esq., M.R.C.S., &c. ... ... ... 213

Stereoscopic X-Ray Representation, with an Example. By William Cotton, M.A., M.D., D.P.H.... ... ... ... ... ... 218

The Treatment of Certain Deformities, more especially of the Nose, by the Subcutaneous Injection of Paraffin. By J. Paul Bush, C.M.G., M.R.C.S. ... ... ... ... ... ... ... 220

CONTENTS.

Page

Notes on Three Pathological Specimens. By C. E. S. Flemming, M.R.C.S., L.R.C.P. ... ... ... ... ... ... ... 222

The Unreliability of the Microscope in the Diagnosis of Malignant Disease. By C. Hamilton Whiteford, M.R.C.S., L.R.C P.... 226

On the Use of Morphia in Uræmia. By T. M. Carter, M.R.C.S., L.R.C.P., and F. H. Edgeworth, M.B., B.Sc., B.A. ... ... 231

The Medical Life. By George Munro Smith, M.R.C.S., L.R.C.P. 239

The Results of Operation in Fifty-Four Cases of Cancer of the Breast. By Charles A. Morton, F.R.C.S. ... ... ... 305

The Indications for Treatment in Nephroptosis. By James Swain, M.S., M D. Lond., F R.C.S. ... ... ... ... ... ... 315

Observations on Cases of Appendicitis. By T. Carwardine, M.S. Lond., F.R.C.S. ... ... ... ... ... ... ... 319

Tubal Abortion. By D. C. Rayner, F.R.C.S.... ... ... ... 323

Vasomotor and Ocular Phenomena in Relation to Rheumatoid Arthritis. By R. Llewelyn Jones, M.B. Lond., M.R.C.S., L.R.C.P ... ... ... ... ... ... ... ... ... 328

Progress of the Medical Sciences ... ... 61, 153, 235, 333

Reviews of Books ... ... ... ... ... 70, 164, 248, 345

Editorial Notes ... ... ... ... ... 76, 176, 270, 364

Notes on Preparations for the Sick... ... 82, 179, 275, 368

The Library of the Bristol Medico-Chirurgical Society ... ... ... ... 83, 182, 277, 372

Meetings of Societies ... ... ... ... 85, 186, 282, 377

Local Medical Notes ... ... ... ... 94, 191, 286, 382

Obituary ... ... ... ... ... ... ... ... ... 191

### Bristol Medico-Chirurgical Society.

---

### PAST PRESIDENTS.

874—75. FREDERICK BRITTAN, M.D., B.A. Dub

1875—76. ROBERT WILLIAM COE.

1876—77. SAMUEL MARTYN, M.D. Ed.

1877—78. AUGUSTIN PRICHARD, M.D. Berlin.

1878—79. HENRY EDWARD FRIPP, M.D. Würzburg.

1879—80. WILLIAM MICHELL CLARKE.

1880—81. JOSEPH GRIFFITHS SWAYNE, M.D. Lond.

1881—82. EDWARD LONG FOX, M.D. Oxon.

1882—83. JAMES GEORGE DAVEY, M.D. St. And.

1883—84. WILLIAM JOHNSTONE FYFFE, M.D., B.A. Dub.

1884—85. GEORGE FORSTER BURDER, M.D. Aberd.

1885—86. WILLIAM HENRY SPENCER, M.D., M.A. Cantab.

1886—87. FRANCIS POOLE LANSDOWN.

1887—88. LEMUEL MATTHEWS GRIFFITHS.

1888—89. ROBERT SHINGLETON SMITH, M.D., B.Sc. Lond.

1889—90. NELSON CONGREVE DOBSON.

1890—91. SAMUEL HENRY SWAYNE.

1891—92. FRANCIS RICHARDSON CROSS, M.B. Lond.

1892—93. EDWARD MARKHAM SKERRITT, M.D., B.S., B.A. Lond.

1893—94. JAMES GREIG SMITH, M.B., C.M., M.A. Aberd., F.R.S.E.

1894—95. ALFRED JAMES HARRISON, M.B. Lond.

1895—96. ARTHUR WILLIAM PRICHARD.

1896—97. ALFRED EDWARD AUST LAWRENCE, M.D., C.M. Aberd.

1897—98. JOHN EDWARD SHAW, M.B., C.M. Ed.

1898—99. ROBERT ROXBURGH, M.B. Ed.

1899—00. WILLIAM HENRY HARSANT.

1900—01. DAVID SAMUEL DAVIES, M.D. Lond.

1902.

### LIST OF SUBSCRIBERS

TO THE

### Bristol Medico-Chirurgical Journal.

*Those marked * are Members of the Society.*

| | |
|---|---|
| ACKLAND, C. H. | Moorland Court, Poole Road, Bournemouth. |
| ACKLAND, J. McKNO | 24 Southernhay, Exeter. |
| *ACKLAND, W. R. | 5 Rodney Place, Clifton. |
| ADYE, W. J. A. | Church House, Bradford, Wilts. |
| AKERS, W. DUTTON | 89 Goldstone Villas, Hove, Sussex. |
| ALDOUS, GEORGE F. | Charlton House, Mannamead, Plymouth |
| ALDRIDGE, CHARLES, M.D., C.M. Aberd. | Plympton. |
| ALEXANDER, JAMES, M.D., M.Ch., B.A. Dub. | Bishop's Place, Paignton. |
| AMBROSE, J., M.B., C.M. Aberd. | 138 Fishponds Road, Eastville, Bristol. |
| AVELINE, H. T. S. | Somerset Asylum, Taunton. |
| *BALLANCE, H. STANLEY, M.D. Lond. | 4 Royal Terrace, Weston-super-Mare. |
| *BARCLAY, W. M. | 21 Oakfield Road, Clifton. |
| BARETTI, T. G. L'ENARDI | 49 Royal York Crescent, Clifton. |
| *BARKER, G. H., M.B., B.Sc. Lond. | 124 Redland Road, Bristol |
| *BARON, BARCLAY J., M.B., C.M. Ed. | 16 Whiteladies Road, Clifton. |
| *BASSET, WALTER | 24 Stow Hill, Newport, Mon. |
| BATTEN, RAYNER W., M.D. Lond. | 1 Brunswick Square, Gloucester. |
| BATTEN, W. SMITH | West Hill, Calne. |
| BAYER Co., LTD. | 20 Booth Street, Mosley Street, Manchester. |
| *BEDDOE, JOHN, M.D. Ed., B.A. Lond., F.R.S. | The Chantry, Bradford-on-Avon. |
| BENHAM, HARRY A., M.D., C.M. Aberd. | Asylum, Fishponds, Gloucestershire. |
| BENNETT, W. S. | Escot, Penzance. |

### LIST OF SUBSCRIBERS.

| | |
|---|---|
| BERNARD, C. | Fishponds. |
| *BERRY, F. C., M.D., B.Ch., B.A. Dub. | Burnham, Somersetshire. |
| BERRY, JAMES, M.B., B.S. Lond. | 21 Wimpole Street, London, W. |
| BIAMONTI, SEBASTIANO | 27 Via Balbi, Genoa, Italy. |
| *BLACKER, A. E. | Richmond Hill Avenue, Clifton. |
| *BLAGG, ARTHUR F., M.D. Durh. | 28 Caledonia Place, Clifton. |
| BOYCE, JAMES G. | The Chipping, Wotton-under-Edge. |
| BOYD, GEO. S. J. | 10 Adelaide Terrace, Portishead. |
| BRAINE-HARTNELL, J. C. R. | Cotswold Sanatorium, Cranham, near Stroud. |
| *BRASHER, CHARLES W. J. | 128 Cotham Brow, Bristol. |
| BRIGGS, H. FIELDEN, D.D.S. Mich. | 6 West Mall, Clifton. |
| *BRISTOWE, H. C., M.D. Lond. | Wrington, Somerset. |
| BROWN, G. ARTHUR | Tredegar. |
| BROWN, WILLIAM, M.D., C.M. Glasg. | Fishponds, Gloucestershire. |
| BROWNE, J. WALTON, M.D., B.A. Royal Univ. I. | 10 College Square North, Belfast. |
| *BULLEN, F. ST. JOHN | 12 Pembroke Road, Clifton. |
| *BUNBURY, E. G. | 13 Dowry Square, Hotwells. |
| BURROUGHS, E. F. H. | 3 Elgin Park, Bristol. |
| *BUSH, J. PAUL, C.M.G. | Vyvyan House, Clifton Park, Clifton. |

| | |
|---|---|
| *CARR, ALEXANDER | 150 Wells Road, Bristol. |
| *CARTER, T. M. | Victoria Square, Clifton. |
| *CARWARDINE, THOMAS, M.B., M.S. Lond. | 16 Victoria Square, Clifton. |
| *CAVE, EDWARD J., M.D. Lond. | 20 Circus, Bath. |
| *CHILTON, R. H. | Tudor House, Cambridge Park, Bristol. |
| CHUTE, HENRY M. | King William's Town, S. Africa. |
| *CLARKE, JOHN MICHELL, M.D., M.A. Cantab. | 28 Pembroke Road, Clifton. |
| *COOK, HENRI, M.D., C.M. Aberd. | 45 Stackpool Road, Bristol. |
| CORNALL, Miss | 20 Arley Hill, Bristol. |
| *CORRIGAN, W. J. | Cloughmore, Splott Avenue, Cardiff. |
| COSSHAM, W. R., M.D., C.M. Aberd. | Cirencester. |
| COTTON, WILLIAM, M.B., C.M., M.A. Ed. | 231 Gloucester Road, Bristol. |
| COULTER, R. J., M.B. Dub. | Warwick House, Stow Hill, Newport, Mon. |
| COUSINS, J. WARD, M.D. Lond. | Kent Road, Southsea. |
| COWAN, F. S. | 27 Queen Square, Bath. |
| CRADDOCK, SAMUEL | South Lyncombe, Bath. |
| *CRIDLAND, A. B. | Bristol. |
| CRISP, J. ELLIS | Alexander House, Corsham. |
| *CROSS, F. RICHARDSON, M.B. Lond. | Worcester House, Clifton. |
| *CROSSMAN, EDWARD, M.D. Dur. | Hambrook, Gloucestershire. |
| *CROSSMAN, F. W. | Hambrook, Gloucestershire. |
| CROUCH, C. PERCIVAL, M.B. Lond. | 3 Princes Buildings, Weston-super-Mare. |
| CULROSS, JAMES, M.B., C.M., M.A. Glasg. | 66 Queen Street Newton Abbot. |

| | |
|---|---|
| *DACRE, JOHN | 14 Eaton Crescent, Clifton. |
| DALBY, AUGUSTUS W. | Redland House, Frome. |
| *DAVIES, D. S., M.D. Lond., D.P.H. Cantab. | 60 Oakfield Road, Clifton. |
| DAVIS, THEODORE, M.D. Lond. | Beachcroft, Clevedon. |
| DAVY, HENRY, M.D. Lond | Southernhay House, Exeter. |
| DENING, EDWIN | Manor House, Stow-on-the-Wold. |

### LIST OF SUBSCRIBERS.

| | |
|---|---|
| DICKIE, JAMES STEEL, M.D., C.M. Aberd. ... | Boscombe Park, Bournemouth. |
| *DOBSON, NELSON C. ... ... ... ... ... ... | Avonmore, Sneyd Park. |
| DOWN, A. R. ... ... ... ... ... ... ... ... | Bampton, Devon. |
| DUNBAR, ELIZA L. WALKER, M.D. Zürich ... | 9 Oakfield Road, Clifton. |

| | |
|---|---|
| EADON, W. F. BAILEY ... ... ... ... ... ... | Hambrook, Gloucestershire. |
| *EAGER, REGINALD, M.D. Lond. ... ... ... ... | Northwoods, Winterbourne. |
| *EAGER, W. R. ... ... ... ... ... ... ... ... | Northwoods, Winterbourne. |
| EBERLE, EMILY, M.A., M.B., B.Ch. Dub. ... | 17 Oakfield Road, Clifton. |
| *EDGEWORTH, F. H., M.B., B.C., B.A. Cantab., B.Sc. Lond. ... ... ... ... ... ... ... ... | 35 Pembroke Road, Clifton. |
| ELDER, WILLIAM ... ... ... ... ... ... ... | 7 Trelawney Road, Bristol. |
| *ELLIOTT, CHRISTOPHER, M.D., B.A. Dub. ... | 88 Pembroke Road, Clifton. |
| ELLIOTT, F. PERCY, M.B., C.M. Aberd. ... ... | 113 Grove Road, Walthamstow, London, N.E. |
| ELLIS, W. McD., M.D. Brux. ... ... ... ... | Cedar Lodge, Bath. |
| ESSEX, J. R. ... ... ... ... ... ... ... ... | Pontypool, Monmouthshire. |
| EVANS, T. G. C. ... ... ... ... ... ... ... | Budleigh Salterton, Devon. |

| | |
|---|---|
| FAIRBANKS, W., M.D., C.M. Ed. ... ... ... ... | Wells, Somersetshire. |
| *FAWCETT, E., M.B., C.M. Ed. ... ... ... ... | 141 Redland Road, Bristol. |
| *FENDICK, R. GEORGE ... ... ... ... ... ... | St. Paul's Road, Clifton. |
| *FIRTH, J. L., M.D., B.S. Lond. ... ... ... ... | 20 Richmond Park Road, Clifton. |
| *FISHER, THEODORE, M.D. Lond.... ... ... ... | Harley Lodge, Clifton. |
| *FLEMMING, A. L. ... ... ... ... ... ... ... | The Asylum, Fishponds. |
| *FLEMMING, C. E. S. ... ... ... ... ... ... | Manvers House, Bradford-on-Avon. |
| *FLETCHER, J., M.B., C.M. Aberd. ... ... ... | Ham Green, Somerset. |
| FLOOD, P. ... ... ... ... ... ... ... ... ... | 318 Stapleton Road, Bristol. |
| FORD, JOSEPH ... ... ... ... ... ... ... ... | Wedmore. |
| FORTY, D. H. ... ... ... ... ... ... ... ... | Wotton-under-Edge. |
| FOSS, E. V. ... ... ... ... ... ... ... ... | Babbacombe House, Ashton Road, Bedminster, Bristol. |
| FOWLER, O. H. ... ... ... ... ... ... ... ... | Cirencester. |
| *FOX, BONVILLE B., M.D., M.A. Oxon. ... ... | Brislington. |
| *FOX, E. LONG, M.D. Oxon. ... ... ... ... ... | 4 Clifton Park, Clifton. |
| FOX, F. F. ... ... ... ... ... ... ... ... ... | Yate House, Chipping Sodbury. |
| FOX, T. COLCOTT, M.B. Lond., B.A. Cantab.... | 14 Harley Street, London, W. |
| FRAZER-TOOVEY, T. E. ... ... ... ... ... ... | The Hospital, Teignmouth. |
| FREEMAN, J., M.D. Brux. ... ... ... ... ... | Glencairn Villa, Queen's Road, Clifton. |

| | |
|---|---|
| GAMBLE, C. H. ... ... ... ... ... ... ... ... | Barnstaple. |
| GARLAND, E. B., M.B., C.M. Edin. ... ... ... | 92 Hampton Road, Redland. |
| GENGE, T. T. ... ... ... ... ... ... ... ... | 21 Whiteladies Road, Bristol. |
| GENT, W. C. ... ... ... ... ... ... ... ... | 170 Wells Road, Bristol. |
| *GERRISH, D. S. ... ... ... ... ... ... ... ... | Cambridge House, Whitehall, Bristol. |
| *GIBBS, ALFRED N. GODBY ... ... ... ... ... | 52 Whiteladies Road, Clifton. |
| GIDLEY, G. G. ... ... ... ... ... ... ... ... | Cullompton, Devon. |
| GOSS, T. BIDDULPH... ... ... ... ... ... ... | 1 Circus, Bath. |
| GRACE, ALFRED ... ... ... ... ... ... ... | Chipping Sodbury. |
| GRACE, EDWARD MILLS... ... ... ... ... ... | Thornbury, Gloucestershire |

| | |
|---|---|
| Gratte, C. Brooke | 183 Commercial Road, Newport, Mon. |
| *Greer, W. J. | 2 Chepstow Road, Newport, Mon. |
| Grey, T. C. | Camborne, Cornwall. |
| *Griffiths, J. S. | 25 Redland Park, Bristol. |
| *Griffiths, L. M. | 11 Pembroke Road, Clifton. |
| Griffiths, P. Rhys, M.B., B.S. Lond. | 71 Newport Road, Cardiff. |
| *Groves, E. W. H., M.B., B.Sc. Lond. | Kingswood, Bristol. |
| Hadwen, W. R., M.D. St. And. | 35 Brunswick Square, Gloucester. |
| *Hailes, Clements D. G., M.D., C.M. Ed. | 27 Alma Road, Clifton. |
| *Haines, A. H. | 8 Cotham Road, Bristol. |
| Hamilton, D. L. | Timsbury, near Bath. |
| Handfield-Jones, Montagu, M.D. Lond. | 35 Cavendish Square, London, W. |
| Harper, Joseph | Bear Street, Barnstaple. |
| *Harris, H. Elwin, B.A., M.B. Cantab. | 13 Lansdown Place, Clifton. |
| *Harrison, A. J., M.B. Lond. | Guthrie Road, Clifton. |
| Harsant, J. G., M.D., B.S. Lond. | Exeter Road, Bournemouth. |
| *Harsant, W. H. | Tower House, Clifton. |
| *Harvey, Alfred, M.B. Lond. | Westbury-on-Trym. |
| Hay, M. B. | North Devon Infirmary, Barnstaple. |
| Hayman, Alfred G. | Hapsford House, near Frome. |
| *Hayman, C. A., M.D. St. And. | Kingston Villa, Richmond Hill, Clifton. |
| Heath, Miss | 7 Gwynn Street, Bristol. |
| *Heaven, John C., D.P.H. Lond. | 17 Whiteladies Road, Bristol. |
| *Herapath, C. K. C. | Cotham Park, Bristol. |
| *Hetling, H. E. | 30 Elliston Road, Bristol. |
| Hill, Hedley | 1 Redcliff Hill, Bristol. |
| *Hill, Thomas, M.D. St. And. | 138 Cromwell Road, Bristol. |
| *Hill, Walter J. | Edgcumbe Villa, Clevedon. |
| Hoffman, A. W. W. | 30 The Parade, Leamington. |
| Horder, T. Garrett | 32 Windsor Place, Cardiff. |
| Hospital, Bristol General (*Sec.*, W. Thwaites). | |
| Hospital, Taunton and Somerset (*House-Surgeon*, W. Drake). | |
| Howells, William, M.B., C.M. Glasg. | Watton House, Brecon. |
| Huggard, W. R., M.D., M.Ch., M.A. Roy. Univ. I. | Davos-Platz, Switzerland. |
| Hunt, A. D. | Millbrook House, Chagford. |
| Imlay, G. A., M.B., C.M. Aberd. | 1 Cotham Park, Bristol. |
| Ingle, C. Durban | Somerton, Somerset. |
| Ives, William R. Y. | 6 Portswood Hill, Bevois Hill, Southampton. |
| Jackson, Mark, M.D. Roy. Univ. I. | Bear Street, Barnstaple. |
| Jacobson, W. H. A., M.B., M.C., M.A. Oxon. | 66 Great Cumberland Place, London, W. |
| Jamison, A., M.B., B.Ch., M.A. Roy. Univ. I. | 66 Woodstock Road, Belfast. |
| Jones, Evan | Ty-mawr, Aberdare. |
| *Jones, E. J. Trevor, M.D. Brux. | Aberdare, Glamorganshire. |
| Jones, H. Macnaughton, M.D., M.Ch. Roy. U.I. | 131 Harley Street, London, W. |
| Jones, W. W., M.B., C.M. Ed. | 47 Wellington Street, Merthyr Tydvil. |
| *Kemm, F. St. J. | Worle. |
| *Kendall, H. W. | 1 Burlington Road, Redland. |

| | |
|---|---|
| KENNEDY, W. P., M.D., B.Ch., B.A. Dub. ... | High Street, Lydney. |
| KINNEIR, R. ... ... ... ... ... ... ... ... ... | The Tower House, Malmesbury. |
| *KYLE, H. G., M.B., B.Ch. Oxon. ... ... ... | 53 Pembroke Road, Clifton. |

| | |
|---|---|
| *LACE, F. ... ... ... ... ... ... ... ... ... ... | 1 Paragon, Bath. |
| LANCASTER, E. LE CRONIER, B.A., M.B., B.Ch. Oxon. ... ... ... ... ... ... ... ... ... ... | Winchester House, Swansea. |
| *LANSDOWN, F. POOLE ... ... ... ... ... ... ... | 19 Whiteladies Road, Clifton. |
| *LANSDOWN, R. G. P., M.B., B.S. Dur. ... ... | 39 Oakfield Road, Clifton. |
| LAWRIE, J. MACPHERSON, M.D., C.M. Glasg.... | Greenhill, Weymouth. |
| LE SOUDIER, H. ... ... ... ... ... ... ... ... | 174 et 176 Boulevard St. Germain, Paris. |
| *LEES, E. LEONARD, M.D., C.M. Ed. ... ... ... | Chantry Road, Clifton. |
| LEIGH, W. W. ... ... ... ... ... ... ... ... ... | Treharris, Glamorganshire. |
| *LEONARD, R. C., M.D. Ed. ... ... ... ... ... | 134 Coronation Road, Bristol. |
| *LIGERTWOOD, WALTER ... ... ... ... ... ... | Bath and Somerset County Asylum, Wells. |
| LINDEN, H. COOKE ... ... ... ... ... ... ... | Compton Martin, Somerset. |
| LINDSAY, JAMES A., M.D., M.Ch., M.A. Roy. U.I. ... ... ... ... ... ... ... ... ... | 37 Victoria Place, Belfast. |
| LISTER, LORD, P.R.S. ... ... ... ... ... ... | 12 Park Crescent, London, N.W. |
| LOWE, T. PAGAN ... ... ... ... ... ... ... | 16 Circus, Bath. |
| *LUCAS, J. J. S. ... ... ... ... ... ... ... ... | Wells Road, Totterdown. |
| LUCY, REGINALD H. ... ... ... ... ... ... | 9 The Crescent, Plymouth. |

| | |
|---|---|
| *McCREA, P. W., M.D., B.Ch., B.A.O., B.A. Dub. ... ... ... ... ... ... ... ... ... ... | 19 Portland Square, Bristol. |
| MACDONALD, P. W., M.D., C.M. Aberd.... ... | County Asylum, Dorchester. |
| McELFATRICK, JOHN ... ... ... ... ... ... ... | Stonycroft Grange, Alderley Edge, Cheshire. |
| *McKEE, A. B., M.B., B.Ch. Dub. ... ... ... | Sussex Villa, Sussex Place, Bristol. |
| *MACKIE, F. P. ... ... ... ... ... ... ... ... ... | Fylton Rectory, near Bristol. |
| MACKINNON, CHARLES, M.B., C.M., M.A. Glasg. | Davaar, Cirencester. |
| MACLEAN, EWEN J., M.D., C.M. Ed.... ... ... | 12 Park Place, Cardiff. |
| MACLEAN, J. CAMPBELL, M.B., C.M. Ed.... ... | Swindon House, Swindon, Wiltshire. |
| MACREADY, J. ... ... ... ... ... ... ... ... | 42 Devonshire Street, Portland Place, London, W. |
| McWATTERS, J. C. ... ... ... ... ... ... | Almondsbury. |
| MARSH, O. E. BULWER ... ... ... ... ... ... | Parkdale, Newport, Mon. |
| MARSHALL, ARTHUR L., M.B., B.A. Cantab. ... | Thornleigh, Upper Clapton, London. |
| *MARTIN, E. F., M.B. Ed. ... ... ... ... ... | Victoria House, Weston-super-Mare. |
| *MARTIN, R. C. ... ... ... ... ... ... ... ... | 3 Clifton Vale, Clifton. |
| *MARTYN, G. J. KING, M.D., B.C., B.A. Cantab. ... ... ... ... ... ... ... ... ... | 12 Gay Street, Bath. |
| MASON, S. B. ... ... ... ... ... ... ... ... ... | 12 Park Terrace, Pontypool. |
| MASON, WILLIAM ... ... ... ... ... ... ... ... | Sedgemoor Cottage, St. Austell. |
| MATTHEWS, CHARLES E., M.D. Dur. ... ... | 6 Hickman Road, Penarth. |
| MATTHEWS, H. E. ... ... ... ... ... ... ... ... | The Mall Pharmacy, Clifton. |
| *MELSOME, W. S., M.D. Cantab.... ... ... ... | 20 Circus, Bath. |
| MERCK, E. ... ... ... ... ... ... ... ... ... ... | 16 Jewry Street, London, E.C. |
| MEREDITH, JOHN, M.D. Ed. ... ... ... ... ... | Wellington, Somersetshire. |

| | |
|---|---|
| *MILLARD, W. J. KELSON, M.D. St. And ... ... | 7 Bayshill Villas, Cheltenham. |
| MITCHELL, TRAFFORD, M.D. ... ... ... ... | Gorseinon, R.S.O., Glamorganshire. |
| *MOLE, HAROLD F. ... ... ... ... ... ... ... ... | Royal Infirmary, Bristol. |
| MONCKTON, WILLIAM ... ... ... ... ... ... ... | Portishead. |
| MORGAN, ALBERT T., M.D. Brux. ... ... ... | 89 Chesterfield Road, St. Andrew's Park, Bristol. |
| MORGAN, JOHN ... ... ... ... ... ... ... ... ... | Langport, Somersetshire. |
| *MORGAN, T. WHITWORTH S. ... ... ... ... | Pill. |
| *MORTON, C. A. ... ... ... ... ... ... ... ... ... | 14 Vyvyan Terrace, Clifton. |
| *MORTON, W. B., M.D. Lond. ... ... ... ... | Brislington House, Brislington. |
| MUNDEN, CHARLES ... ... ... ... ... ... ... ... | Ilminster. |
| MUZUMIDAR, NOGENDRANATH, M.B.... ... ... | Bhagalpore, Bengal, India. |
| *MYLES, G. T. ... ... ... ... ... ... ... ... ... | 147 Whiteladies Road, Bristol. |

| | |
|---|---|
| *NEILD, NEWMAN, M.B., B.Ch. Vict. ... ... ... | Richmond Hill, Clifton. |
| *NEVILL, W. N., M.D., B.Ch., B.A. Dub. ... ... | Leighton Villa, Southville, Bristol. |
| *NEWMAN, CHARLES ... ... ... ... ... ... ... ... | 156 Cheltenham Road, Bristol. |
| *NEWNHAM, W. H. C., M.B., M.A. Cantab. ... | Chandos Villa, Clifton. |
| NICHOLSON, T. D., M.D. Ed. ... ... ... ... | 2 Whiteladies Road, Clifton. |
| NORMAN, J. H. ... ... ... ... ... ... ... ... ... | Goodleigh, Winkleigh, Devonshire. |

| | |
|---|---|
| O'BRIEN, CORNELIUS ... ... ... ... ... ... ... | Devon House, Whitehall, Bristol. |
| ODELL, WILLIAM ... ... ... ... ... ... ... ... | Ferndale, Torquay. |
| *OGILVY, ALEXANDER, M.D., B.Ch., B.A. Dub. | Montrose House, Queen's Road, Clifton. |
| OPENSHAW, E. H. ... ... ... ... ... ... ... ... | Cheddar. |
| *ORMEROD, H. LAWRENCE, M.B., B.Ch., B.A.O., R.U.I. ... ... ... ... ... ... ... ... ... ... | Burnley, Westbury-on-Trym |

| | |
|---|---|
| PAGE, G. SHEPLEY ... ... ... ... ... ... ... ... | 78 Old Market Street, Bristol. |
| *PARKER, GEORGE, M.D., M.A. Cantab. ... ... | 14 Pembroke Road, Clifton. |
| PARRY, J. H. ... ... ... ... ... ... ... ... ... | Ashley Road, Bristol. |
| PARSONS, FRANCIS J. C.... ... ... ... ... ... | Ivy House, Bridgwater. |
| *PARSONS, H. F.... ... ... ... ... ... ... ... ... | The Heath, Stoke Bishop. |
| PATERSON, DONALD ROSE, M.D., C.M. Ed. ... | 18 Windsor Place, Cardiff. |
| PEAKE, ARTHUR W.... ... ... ... ... ... ... ... | 34 Gloucester Road, Bishopston, Bristol. |
| PEAKE, G. ARTHUR ... ... ... ... ... ... ... ... | Rodney Place, Cheltenham. |
| *PEARSE, E. M. ... ... ... ... ... ... ... ... ... | 51 Claredon Road, Redland, Bristol. |
| PECK, Major, R.A.M.C. ... ... ... ... ... ... | United Service Club, Calcutta. |
| *PERRY, W. A. ... ... ... ... ... ... ... ... ... | Livorno, Rushton Crescent, Bournemouth. |
| *PHILLIPS, C. M., M.D. Brux. ... ... ... ... | Ravenslea, Kensington Hill, Brislington. |
| PHILLIPS, EDWARD PICTON ... ... ... ... ... | High Street, Haverfordwest. |
| *PICKERING, C. F. ... ... ... ... ... ... ... ... | 13 Berkeley Square, Bristol. |
| PLAISTER, W. H. ... ... ... ... ... ... ... ... | High Road, Tottenham, N. |
| POLLARD, REGINALD, M.B. Dur. ... ... ... | 8 Higher Terrace, Torquay. |
| *POUNTNEY, W. E., M.D., C.M. Ed. ... ... ... | 33a Whiteladies Road, Bristol. |
| POWELL, J. J., M.D. Lond. ... ... ... ... ... | 2 Portland Square, Bristol. |

| | |
|---|---|
| *Powell, L. W. ... ... ... ... ... ... ... ... ... | Two Mile Hill Road, Kingswood, Bristol. |
| Powell, William, M.B. Lond. ... ... ... ... ... | Hill Garden, Torquay. |
| *Prichard, Arthur W. ... ... ... ... ... ... ... | 6 Rodney Place, Clifton. |
| *Prowse, Arthur B., M.D. Lond. ... ... ... | 5 Lansdown Place, Clifton. |
| Pruen, S. T., M.D. Dur. ... ... ... ... ... ... | Sherborne Lodge, Cheltenham. |
| Purdy, J. R., M.D. Aber. ... ... ... ... ... ... | Lower Hutt, New Zealand. |

| | |
|---|---|
| Randolph, Charles ... ... ... ... ... ... ... | St. Michael's, Milverton, Somersetshire. |
| *Rattray, J. M., M.D., C.M., M.A. Aberd. ... | Frome. |
| *Rayner, D. C. ... ... ... ... ... ... ... ... ... | 7 Victoria Square, Clifton, Bristol. |
| Redwood, T. Hall, M.D., C.M., M.A.Dur. ... | The Lawn, Rhymney, Monmouthshire. |
| Rendall, John ... ... ... ... ... ... ... ... | Forestside, Lymington. |
| Riddell, Andrew W. ... ... ... ... ... ... ... | New Brighton, Cheshire. |
| Roberts, Frederick T., M.D., B.Sc. Lond. | 102 Harley Street, London, W. |
| Roderick, Sydney J., M.B., C.M. Ed. ... ... | Llanelly. |
| Rodgers, John W., M.B., C.M. Ed. ... ... | Redfield, Gloucestershire. |
| *Rogers, B. M. H., M.D., B.Ch., B.A. Oxon. ... | 11 York Place, Clifton. |
| *Rose, F. H. ... ... ... ... ... ... ... ... ... | Westfield Park, Clifton. |
| *Rossiter, George F., M.B. Lond. ... ... | Cairo Lodge, Weston-super-Mare. |
| *Roué, W. Barrett, M.D., M.S. Dur. ... ... | 2 Buckingham Place, Clifton. |
| Routh, R. Henry F. ... ... ... ... ... ... ... | Bridgwater. |
| *Roxburgh, Robert, M.B. Ed. ... ... ... | 1 Victoria Buildings, Weston-super-Mare |
| *Rudge, C. King ... ... ... ... ... ... ... ... | 145 Whiteladies Road, Bristol. |
| *Rudge, H. T. ... ... ... ... ... ... ... ... ... | 5 Colston Parade, Bristol. |
| Ruffer, M. Armand, M.D., B.S., M.B. Oxon. | Alexandria, Egypt. |
| Rutherford, H. T., M.A., M.D. Camb. ... ... | Taunton. |

| | |
|---|---|
| Scott, Richard J. H. ... ... ... ... ... ... ... | 28 Circus, Bath. |
| Searle, George C. ... ... ... ... ... ... ... | Brixham, Devonshire. |
| Searle, Richard Burford... ... ... .. ... | Bexley, Dartmouth. |
| *Semple, W. R. M., M.B., C.M. Glasg. ... ... | Beltrees, Yeovil, Somersetshire. |
| Seward, W. J., M.B. Lond. ... ... ... ... | Asylum, Colney Hatch, London, N. |
| *Shaw, J. E., M.B., C.M. Ed. ... ... ... ... | 23 Caledonia Place, Clifton. |
| Shaw, Jesse, M.D.... ... ... ... ... ... ... | Fort Beaufort, Cape Colony. |
| Shaw-Mackenzie, John A., M.D. Lond. ... | 31 Grosvenor Street, Grosvenor Square, W. |
| Sheen, A., M.D. St. And. ... ... ... ... ... | 23 Newport Road, Cardiff. |
| *Sheppard, E. J. .. ... ... ... .. ... ... | 8 Richmond Hill, Clifton. |
| *Shoolbred, W. A. ... ... ... ... ... ... ... ... | St. Ann's, Chepstow. |
| Simons, R. J. ... ... ... ... ... ... ... ... | Nolton Court, Bridgend. |
| Skelton, Henry, M.D. St. And. ... ... ... | Downend, Gloucestershire. |
| *Skerritt, E. Markham, M.D., B.S., B.A.Lond. | Richmond Hill, Clifton. |
| *Smith, G. Munro ... ... ... ... ... ... ... ... | 18 Apsley Road, Clifton. |
| Smith, Rev. Reginald, M.A. Cantab. ... ... | The Vicarage, Walpole St. Andrew, Wisbech, Norfolk. |
| *Smith, R. Shingleton, M.D., B.Sc. Lond. ... | Clifton Park, Clifton. |
| Smith, W. Alexander, M.B., M.A. Oxon. ... | Newport, Essex. |
| *Smith, W. A. L., M.A., M.B., B.C. Cantab. ... | 8 Chamberlain Street, Wells. |
| Smyth, H. J. ... ... ... ... ... ... ... ... ... | New Road, South Molton. |
| Smyth, J. C. ... ... ... ... ... ... ... ... ... | Wells Road, Glastonbury. |
| Society, Cardiff Medical (*Hon. Lib.*, Queen Street, Cardiff). | |
| Society, Manchester Medical ... ... ... | Medical Society's Library, The Owens College, Manchester. |
| Society, Plymouth Medical (*Hon. Lib.*, J. Elliott Square). | |
| Solly, Reginald V., M.B., B.S. Lond. ... ... | 40 Southernhay, Exeter. |
| Solly, S. E. ... ... ... ... ... ... ... ... ... | Colorado Springs, Colorado, U. S. A. |

| | |
|---|---|
| *Stack, E. H. E., B.A., M.B., B.C. Cantab. ... | Royal Infirmary, Bristol. |
| Stamp, W. D. ... ... ... ... ... ... ... ... | Ridgeway House, Plympton. |
| Staple, J. D. ... ... ... ... ... ... ... ... | ClevedonVilla,Cheltenham Road,Bristol. |
| Statham, R. Whiteside ... ... ... ... ... | Cheddar, Somersetshire. |
| *Steele, Charles, M.D. Dur. ... ... ... ... | 17 Richmond Hill, Clifton. |
| *Steele, W. H., Lt.-Col., M.D. Dub. ... ... | 18 Mortimer Road, Clifton. |
| *Stephenson, J. B., M.B., B.S. Dur. ... ... | 19 Park Row, Bristol. |
| Stevens, W. H. ... ... ... ... ... ... ... | Zetland Road, Bristol. |
| *Stewart, J., B.A., R.U.I. ... ... ... ... ... | Dunmurry, Sneyd Park, Bristol. |
| Stock, P. G., R.A.M.C.... ... ... ... ... ... | c/o P.M.O., South Africa. |
| *Stock, W. S. V., M.B., B.S. Lond. ... ... ... | Royal Infirmary, Manchester. |
| Stoddart, F. Wallis ... ... ... ... ... ... | Southey House, College Green, Bristol. |
| *Swain, James, M.D., M.S. Lond. ... ... ... | 4 Victoria Square, Clifton. |
| *Swayne, J. G., M.D. Lond. ... ... ... ... ... | 74 Pembroke Road, Clifton. |
| *Swayne, W. Carless, M.D. Lond. ... ... ... | St. Paul's Road, Clifton. |
| *Symes, J. O., M.D. Lond. ... ... ... ... ... | 11 Richmond Hill, Clifton. |
| Symonds, H. P. ... ... ... ... ... ... ... | 35 Beaumont Street, Oxford. |
| Symons, John ... ... ... ... ... ... ... ... | Buriton House, Penzance. |
| *Symons, W. H., M.D. Brux.... ... ... ... ... | Guildhall, Bath. |
| | |
| Taylor, C. Bell, M.D. Edin. ... ... ... ... | 9 Park Row, Nottingham. |
| *Taylor, James ... ... ... ... ... ... ... ... | 36 Alma Road, Clifton. |
| *Temple, G. H., M.B., C.M. Ed. ... ... ... | Weston-super-Mare. |
| Theed, S. Vipan ... ... ... ... ... ... ... | Eversfield House, Warminster. |
| Thomas, A. Garrod, M.D., C.M. Ed. ... ... | Clytha Park, Newport, Mon. |
| Thomas, J. Lynn ... ... ... ... ... ... ... | 28 Charles Street, Cardiff. |
| Thomas, Raglan, M.D. Lond. ... ... ... ... | 13 West Southernhay, Exeter. |
| Thomson, Eustace B., M.D., C.M. Glasg. ... | Albany Place, Plymouth. |
| *Tivy, W. J.... ... ... ... ... ... ... ... ... | 8 Lansdown Place, Clifton. |
| Tosswill, Louis H., M.B., B.A. Cantab.... ... | 1 West Southernhay, Exeter. |
| Trevithick, Edgar, M.A., M.D. Cantab. ... | 24 Promenade, Cheltenham. |
| Tyley, R. P., M.D. St. And.... ... ... ... ... | Wedmore. |
| | |
| Vachell, Herbert R., M.D., C.M. Aberd. ... | 18 Newport Road, Cardiff. |
| Vereker, R. H. ... ... ... ... ... ... ... | Eastleigh, Curry Rivel, Somerset. |
| | |
| *Waldo, Henry, M.D., C.M. Aberd. ... ... ... | 19 Pembroke Road, Clifton. |
| *Walker, Cyril H., M.B., B.A. Cantab. ... ... | 16 Pembroke Road, Clifton. |
| Wallace, Rev. Canon, M.A. Oxon. ... ... ... | 3 Harley Place, Clifton. |
| Wallace, J., M.B. ... ... ... ... ... ... ... | 112 York Road, Bedminster |
| *Wallace, John ... ... ... ... ... ... ... | 1 Oriel Terrace, Weston-super-Mare. |
| Walsh, Leslie H., M.B., B.S. Durh. ... ... | 21 Gay Street, Bath. |
| Ward, J. L. W.... ... ... ... ... ... ... ... | Victoria Street, Merthyr Tydvil. |
| *Wathen, J. Hancocke ... ... ... ... ... ... | 16 York Place, Clifton. |
| Watters, George T. B., M.D., C.M. Ed. ... | Stonehouse, Gloucestershire. |
| Weatherly, Lionel A., M.D., C.M. Aberd. | Bailbrook House, Bath. |
| Webb, J. B. ... ... ... ... ... ... ... ... | 10 Upper Barton Hill Road, Barton Hill, Bristol. |
| Webber, William W. ... ... ... ... ... ... | Crewkerne. |
| Whitby, C. J., B.A., M.D. Cantab. ... ... ... | Wynnstay Sanatorium, Burgess Hill, Sussex. |
| White, J. W. ... ... ... ... ... ... ... ... | Orchard House, Nailsea. |
| Wickham, W. ... ... ... ... ... ... ... ... | Hill House, Tetbury. |
| Wigan, C. A., M.D. Dur. ... ... ... ... ... | Portishead. |
| Wigmore, J., M.D. Roy. Univ. I. ... ... ... | 20 Green Park, Bath. |
| Wilcox, Henry, M.B. Aberd. ... ... ... ... | Fleet, Hampshire. |

| | |
|---|---|
| Wilkin, Griffith C. ... ... ... ... ... ... | West Coker, Somerset. |
| Willcox, R. Lewis ... ... ... ... ... ... | Warminster. |
| Willett, George G. D. ... ... ... ... ... | Keynsham, Somersetshire. |
| *Williams, E. C., M.B., B.C., B.A. Cantab. ... | 9 Whatley Road, Clifton. |
| Williams, H. Egerton ... ... ... ... ... | The Mount, Newport, Mon. |
| *Williams, Lionel H. ... ... ... ... ... ... ... | Thornbury, Gloucestershire. |
| *Williams, P. Watson, M.D. Lond.... ... ... | 1 Victoria Square, Clifton. |
| Williams, Peter ... ... ... ... ... ... ... ... | Ferryside, Carmarthenshire. |
| *Williams, W. Roger ... ... ... ... ... ... | Beaufort House, Clifton Down, Clifton. |
| Willis, W. M.... ... ... ... ... ... ... ... ... | 24 Park Street, Nottingham. |
| *Wills, W. K., M.B., B.C. Camb. ... ... ... | 59 Apsley Road, Clifton. |
| *Windsor-Aubrey, H. W. ... ... ... ... ... | Coronation Road, Bristol. |
| *Wintle, Colston ... ... ... ... ... ... ... ... | 24 Gloucester Road, Bristol. |
| *Wohlmann, A. S., M.D., B.S. Lond. ... ... | 9 Gay Street, Bath. |
| Wood, C. R., M.A., M.D. Durh. ... ... ... ... | Bridge House, Frome. |
| Woodhead, G. Sims, M.D., C.M. Ed. ... ... | Pathological Laboratory, New Museum, Cambridge. |
| Woollcombe, Walter L. ... ... ... ... ... | 16 The Crescent, Plymouth. |
| *Young, J., M.D., C.M. Glasg. ... ... ... ... | Clouds Hill House, St. George, Bristol. |

### The Library of the Bristol Medico-Chirurgical Society.

*The following donations have been received since the publication of the List in December:*

*February 28th, 1902.*

| | |
|---|---|
| J. Paul Bush, C.M.G. (1)... ... ... ... ... ... ... | 3 volumes |
| L. M. Griffiths (2) ... ... ... ... ... ... ... ... | 6 ,, |
| The Laryngological Society of London (3) ... ... | 1 volume. |
| The Pathological Society of London (4) ... ... | 1 ,, |
| The Surgeon-General, United States Army (5) ... | 1 ,, |
| James Swain, M.D. (6) ... ... ... ... ... ... ... | 1 ,, |

A framed picture has been presented by Mr. C. E. S. Flemming.

### FORTY-THIRD LIST OF BOOKS.

The titles of books mentioned in previous lists are not repeated.

The figures in brackets refer to the figures after the names of the donors, and show by whom the volumes were presented. The books to which no such figures are attached have either been bought from the Library Fund or received through the *Journal*.

**Bailey, W. C.** ... *The Lepers of our Indian Empire* ... ... ... ... (2) [1891]
**Brunton, Sir L.** ... *On Disorders of Assimilation, Digestion, etc.* ... ... 1901
**Bury, J. S.** ... ... *The Bradshaw Lecture on Prognosis in Relation to Diseases of the Nervous System* ... ... ... ... [N.D.]
*Catalogue of the Library of the Ophthalmological Society of the United Kingdom* ... .. ... ... ... ... ... 4th Ed. 1901
*Catalogue (Index) of the Library of the Surgeon-General's Office, United States Army* ... ... ... (5) 2nd Ser., Vol. VI. 1901
**Chapman, H. J.**... *On Ulcers* ... ... ... ... ... ... ... ... (2) 2nd Ed. 1853
**Cotterell, E.** ... *The Pocket Gray* (Ed. by C. H. Fagge) ... 5th Ed. 1901
**Crile, G. W.** ... *Problems relating to Surgical Operations* ... ... ... 1901
**Cross, F. R.** ... *Some Landmarks in the Progress of Medical Science* ... 1901
**Davidson, A.** ... *Syllabus of Lectures to Nurses* ... ... ... ... ... [N.D.]
**Ellis, H.** ... ... *Studies in the Psychology of Sex—Sexual Inversion* [2nd Ed.] 1901
**Emery, W. D'E.** *Bacteriological Diagnosis* ... ... ... ... ... ... 1902
**Hardy, H. N.** ... *The State of the Medical Profession in Great Britain and Ireland in 1900* ... ... ... ... ... ... (1) 1901
**Haydn, [J.]**... ... *Dictionary of Dates* ... ... ... ... ... 22nd Ed. 1898
**Hollander, B.** ... *The Mental Functions of the Brain* ... ... ... ... 1901
**Hughes, A. W.** ... *Practical Anatomy.* Part II. (Ed. and completed by A. Keith) ... ... ... ... ... ... ... ... 1902
*Medical Tracts* ... ... ... ... ... ... ... ... ... ... ... ... ... ... [V.D.]
**Murray, W.** ... *Rough Notes on Remedies* ... ... ... ... 4th Ed. 1901
**Neff, M. L.**... ... *Prescription Writing* ... ... ... ... ... ... ... 1901
**Osler, W.** ... ... *The Principles and Practice of Medicine* ... 4th Ed. 1901
**Parsons, J. H.** ... *Elementary Ophthalmic Optics* ... ... ... ... ... 1901
*Pasteur, The Life of.* By R. Vallery-Radot. (Tr. by Mrs. R. L. Devonshire) 2 vols. ... ... ... ... ... ... ... 1902
*Physic and Physicians.* 2 vols.... ... ... ... ... ... ... ... ... ... (2) 1839
**Pye, W.** ... ... *Elementary Bandaging and Surgical Dressing.* 9th Ed. (Ed. by T. Carwardine) ... ... ... ... .. 1901
**Rotch, T. M.** ... *Pediatrics* ... ... ... ... ... ... ... ... ... 3rd Ed. 1901
*Selected Essays and Monographs*—English ... ... ... ... ... ... ... 1901
**Semon, Sir F.** ... *Diseases of the Upper Air Passages* ... ... ... ... 1902
**Stirling, W.** ... *Outlines of Practical Physiology* ... ... ... 4th Ed. 1902
**Vallery-Radot, R.** *The Life of Pasteur* (Tr. by Mrs. R. L. Devonshire) 2 vols. ... ... ... ... ... ... ... ... ... ... 1902
**Willis [T.]** ... ... *The London Practice of Physick* ... ... ... ... (2) 1685
,, ... ... *On Preserving those that are well from the Infection of the Plague* ... ... ... ... ... ... ... ... (2) 1691

### TRANSACTIONS, REPORTS, JOURNALS, &c.

American Laryngological Association, Transactions of the ... ... ... 1901
American Ophthalmological Society, Transactions of the Vol. IX., Part II. 1901

American Surgical Association, Transactions of the... ... Vol. XIX. 1901
British Almanac, The ... ... ... ... ... ... ... ... ... ... ... ... ... ... 1902
British Medical Journal, The ... ... ... ... ... ... ... ... Vol. II. for 1901
Catalogue of Books, The English, for 1901... ... ... ... ... ... ... ... 1902
Edinburgh Medical Journal, The ... ... ... ... .. ... N.S., Vol X. 1901
Epidemiological Society of London, Transactions of the N.S., Vol. XX. 1901
Hazell's Annual ... ... ... ... ... ... ... ... ... ... ... ... ... ... 1902
Hospital, The ... ... ... .. ... ... ... ... ... .. Vol. XXX. 1901
Hospitals—
Boston City Hospital, Medical and Surgical Reports of the (6) 1901
St. Bartholomew's Hospital Reports ... ... Vol. XXXVII. 1902
Lancet, The... ... ... ... ... ... ... ... ... ... ... ... Vol. II. for 1901
Laryngological Society of London, Proceedings of the (3) Vol. VIII. 1901
Library Association Record, The ... ... ... ... ... ... ... ... Vol. II. 1900
Medical Directory, The ... ... ... ... ... ... .. ... ... ... ... 1902
Medical Record ... ... ... ... ... ... ... ... ... ... ... Vol. LIX. 1901
Medical Society of London, Transactions of the ... ... Vol. XXIV. 1901
New Hampshire Medical Society, Transactions of the ... ... ... ... 1901
Nordischen Kongresses für innere Medicin zuo Kopenhagen, 1900, Verhandlungen des Dritten ... ... ... ... ... ... ... ... .. 1901
Ohio State Medical Society, Transactions of the ... ... ... ... ... 1901
Ophthalmological Society of the United Kingdom, Transactions of the Vol. XXI. 1901
Otological Society of the United Kingdom, Transactions of the Vol. II. 1901
Pathological Society of London, Transactions of the (4) Vol. LII. 1901
Philadelphia Medical Journal, The ... ... ... ... ... Vol. VII. 1901
Progressive Medicine ... ... ... ... ... ... ... ... Vols. III., IV. 1901
Public Health ... .. ... ... ... ... ... ... ... ... ... Vol. XIII. 1901
Quarterly Medical Journal, The ... ... ... ... ... ... Vol. IX. 1900—01
Smithsonian Institution, Annual Report of the, for 1900... ... ... ... 1901
Whitaker's Almanack ... ... ... ... ... ... ... ... ... ... ... ... 1902
Who's Who... ... ... ... ... ... ... ... ... ... ... ... ... ... ... 1902

### MEETINGS OF SOCIETIES.

### Bristol Medico-Chirurgical Society.

J. Lacy Firth,

*Reporter.*

J. Paul Bush,

*Hon. Sec.*

### Local Medical Notes.

**University College, Bristol.**—Dr. Walter C. Swayne has been appointed Professor of Midwifery. Mr. D. C. Rayner, F.R.C.S., has been appointed Lecturer on Practical Midwifery. In the recent M.D. Examination of the University of London, A. S. Cobbledick, B.S., M.R.C.S., was a successful candidate. Mr. P. White has obtained the M.R.C.S. and L.R.C.P. Diplomas. Mr. H. James has been appointed House Surgeon to the Swansea Hospital.

**Bristol Royal Infirmary.**—E. M. Pearse, M.R.C.S., L.R.C.P., has been appointed Resident Obstetric Officer, and J. C. Clayton, M.R.C.S., L.R.C.P., Junior House Surgeon and Anæsthetist.

**Bristol General Hospital.**—C. F. Pickering, F.R.C.S., has been appointed Consulting Surgeon, J. Lacy Firth, M.D., M.S., F.R.C.S., Surgeon, and H. Greville Kyle, B.A., M.B., B.Ch. (Oxon), Assistant Surgeon to the Bristol General Hospital.

**Royal United Hospital, Bath.**—George Henry Terry, F.R.C.S. Ed., and Frederick Lace, F.R.C.S. Eng., have been appointed Honorary Surgeons, and Harry Lurgan Brownlow, F.R.C.S. Eng., and William H. Cooke, F.R.C.S. Ed., Honorary Assistant-Surgeons, to the Royal United Hospital, Bath.

**Bristol Dispensary.**—The annual report for 1901 of this charitable institution is to hand. It shows that there has been an increase in the number of patients treated above that of last year. During the summer months the Medical Officers had to contend with a severe epidemic of diphtheria, happily with few deaths. The Committee had to deplore the great lack of fresh subscribers to the institution. The Bedminster Branch was continuing to do very good work, and the number of patients treated there was steadily increasing. For this reason the Committee would be grateful for new subscriptions

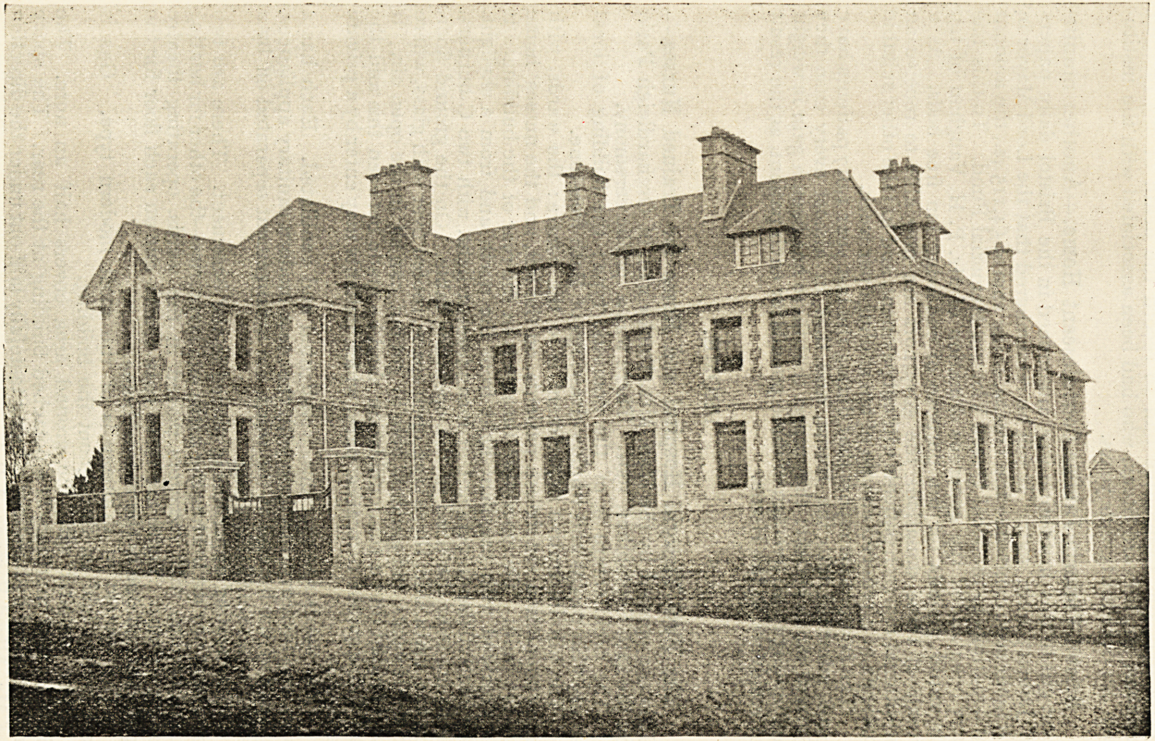

THE NEW VICTORIA HOSPITAL, FROME.

from persons carrying on business in that district. It was with much regret that the Committee had to record the death of one of their number, Mr. Edward Stock, who had been associated with the institution for over thirty-four years.

**Bristol Victoria Jubilee Convalescent Home.**—Another munificent gift to this institution was announced in the Bristol daily papers of January 20th, 1902. It will be remembered that the Lord Mayor in October last remitted to the Treasurer the sum of £4,115 4s., the amount of donations to the local Queen Victoria Memorial Fund. To this has now been added the generous donation of Mr. P. H. Vaughan of ten thousand pounds for the purpose of endowing ten free beds. As a result of these donations the governors are now able to receive an additional fourteen patients without payment, and to take other suitable patients at a reduced charge of ten shillings per week.

The Secretaries report that during the last year 1,017 patients have been admitted into the Home, and that in almost every case they derived much benefit: they report that there has been a gain in weight amounting to an average of three pounds for each patient, as the result of residence in the Home.

### FROME.

**The New Victoria Hospital and Home for Trained Nurses.**—This was opened by the Hon. Mrs. Duckworth, of Orchardleigh Park, on the 12th September, 1901. There was a public ceremony.

The architecture is of the domestic type; the building of local stone, with coigns and window-dressings of Bath freestone.

The administrative department is in the centre: the wards, with accommodation for sixteen patients, on the left, and the nurses' home on the right. The operating theatre is in the rear of the building. It is provided with excellent light from the north and from a skylight. A counterbalance pendant of incandescent light, with two C-burners, is swung over the head of the operating table. On the ground floor, on the south side, there is an open space, roofed over with glass, where convalescents and patients able to get out can sit in the open air. It is intended that a communication shall be made between this and the adjoining public recreation park, which patients may enter by a private gateway.

The wards are spacious, well-lighted and ventilated. Each has a w.c. and lavatory, fitted up with the most modern sanitary appliances, placed at the far end of the ward, the arrangement separated off by a small cross-ventilated space.

The heating apparatus is placed in the basement. There are two systems: one for heating the baths and lavatories—the other for heating the building, with radiators placed in the wards, operating theatre and passages. There are three bath-rooms, one on each floor for the patients and one in the nurses' home.

The system of drainage is of the most modern type. Gas is completely laid on throughout the building.

The hospital with the ground area covers half an acre. The laundry and mortuary is a detached building at the rear. The space not occupied by buildings is being tastefully laid out under the direction of Mr. Alfred Parsons, A.R.A.

The architect of the building is Mr. J. Vaughan Johnson, of London.

